# Supplementary material for: Effect of Shearing and Heat Milling Treatment Temperature on the Crystallinity, Thermal Properties, and Molecular Structure of Rice Starch
Source: Foods. 2023 Mar 1;12(5):1041. doi: 10.3390/foods12051041 (PMC10001028; doi:10.3390/foods12051041)
Supplement: Supplementary file 1 [file foods-12-01041-s001.zip › Supplementary Figure S1.pdf]

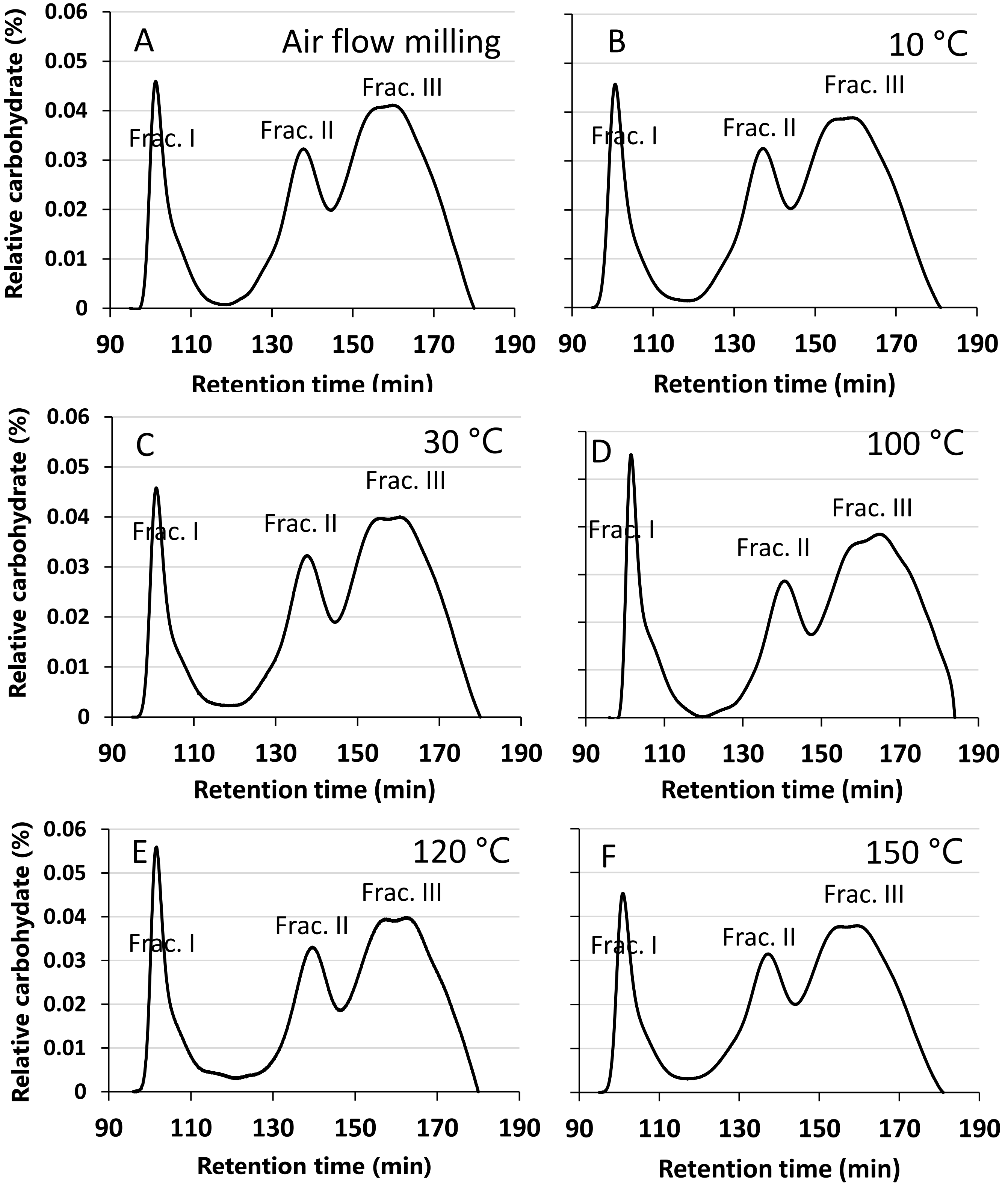

Supplementary Figure S1. Typical patterns of GPC (Toyopearl HW55S-50S × 3) of isoamylase-debranched starch from each rice flour.

Amylose, Amylopectin long chains and Amylopectin short chains were mainly eluted in Fraction (Frac.) I, II and III, respectively .

(A) rice flour using air flow milling, SHMM treatment with different temperature, (B) 10 ℃, (C) 30 ℃, (D) 100 ℃, (E) 120 ℃, and (F) 150 ℃.
