# Supplementary material for: Effect of Shearing and Heat Milling Treatment Temperature on the Crystallinity, Thermal Properties, and Molecular Structure of Rice Starch
Source: Foods. 2023 Mar 1;12(5):1041. doi: 10.3390/foods12051041 (PMC10001028; doi:10.3390/foods12051041)
Supplement: Supplementary file 1 [file foods-12-01041-s001.zip › Supplementary Table S1.pdf]

Supplementary Table S1. Persentage of each peak in GPC (Toyopearl HW75S×2-HW65S-HW55S) of undegraded starch for each rice flour.

		Peak 1 (%)	Peak 2 (%)	Peak 3 (%)
Air flow milling		43.2±0.9 a	41.2±2.2 b	15.6±1.4 c
SHMM	10 °C	46.2±0.9 a	38.7±1.3 b	15.1±0.6 c
	30 °C	32.2±0.5 b	50.9±1.1 ab	16.2±0.6 c
	100 °C	6.3±0.2 c	65.7±0.8 a	28.0±0.6 b
	120 °C	1.1±0.2 cd	52.3±0.9 ab	46.6±0.9 a
	150 °C	0.2±0.1 d	56.0±0.8 ab	43.8±0.9 a

Average±SE, n=3.

The percentage indicate the ratio of each peak to the total carbohydrate (%).

High molecular amylopectin, Low molecular amylopectin and amylose were mainly eluted in Peak 1, 2 and 3, respectively.

Different alphabets in the same column indicate significant differences by Tukey-Kramer method ($p < 0.05$).
